# Clinical, Immunological, and Molecular Findings in Four Cases of B Cell Expansion With NF-κB and T Cell Anergy Disease for the First Time From India

**DOI:** 10.3389/fimmu.2018.01049

**Published:** 2018-06-14

**Authors:** Maya Gupta, Jahnavi Aluri, Mukesh Desai, Madhukar Lokeshwar, Prasad Taur, Michael Lenardo, Jenna Bergerson, Aparna Dalvi, Snehal Mhatre, Manasi Kulkarni, Priyanka Kambli, Manisha Madkaikar

**Affiliations:** ^1^Department of Pediatric Immunology and Leukocyte Biology, National Institute of Immunohaematology (ICMR), Mumbai, India; ^2^Division of Immunology, Bai Jerbai Wadia Hospital for Children, Mumbai, India; ^3^Kashyap Nursing Home, Mumbai, India; ^4^Clinical Genomics Program, Molecular Development of the Immune System Section, Laboratory of Immune System Biology, National Institute of Allergy and Infectious Diseases, National Institutes of Health, Bethesda, MD, United States

**Keywords:** *CARD11*, polyclonal B cell lymphocytosis, NF-κB, GOF mutation, autoimmune lymphoproliferative syndrome

## Abstract

B cell expansion with NF-κB and T cell anergy (BENTA) is a rare primary immunodeficiency disorder caused by mutations in the *CARD11* gene and results in constitutive NF-κB activation in B and T cells. Affected patients present with polyclonal expansion of B cells at an early age with splenomegaly, lymphadenopathy, and mild autoimmunity. Here, we discuss four BENTA cases with unusual clinical manifestations not previously reported. All patients showed previously reported gain-of-function mutations (G123S, G123D, and C49Y) in the *CARD11* gene. Severe autoimmune manifestations were noted for the first time in all our patients.

## Introduction

B cell expansion with NF-κB and T cell anergy (BENTA) disease is a rare form of primary immunodeficiency disorder that has been recently included under the category of “Predominantly antibody deficiencies” by the International Union of Immunological Societies (IUIS) (1).

The disease results from germline heterozygous gain-of-function mutation in the *CARD11* gene. Patients usually present with lymphadenopathy and splenomegaly in early childhood. Impaired T cell proliferation is observed in these patients that increases their susceptibility to recurrent sinopulmonary infections and also to certain opportunistic viral infections like Molluscum contagiosum, BK virus, and Epstein–Barr virus (2). BENTA patients have clinical manifestations that overlap with autoimmune lymphoproliferative syndrome (ALPS) along with marginally elevated double negative T cells (DNT). Significant B cell lymphocytosis and polyclonal expansion of both immature transitional (CD10^+^CD24^hi^CD38^hi^) and mature naïve (IgD^+^) polyclonal B cells, with normal T cell numbers are important clues for the diagnosis of BENTA disease which can be confirmed by genetic analysis. Patients with persistent lymphocytosis and splenomegaly should be evaluated for BENTA disease along with ALPS. Currently, most patients with BENTA disease are closely monitored for B cell neoplasms or infections and managed with minimal intervention. However, application of a targeted therapeutic approach involving the CARD11–BCL10-MALT1 (CBM) signaling pathway or drugs like lenalidomide which are used for diffuse large B cell lymphomas (DLBCL) and MALT1 protease inhibitors may help in the better management of BENTA patients in the near future.

Here, we describe four BENTA cases that have been clinically, immunologically, and molecularly characterized for the first time from India.

## Materials and Methods

### Patients and Samples

The four patients described in this report were diagnosed with BENTA disease at National Institute of Immunohaematology (NIIH). Informed consent for participating in the study was procured from the family members in accordance with the declaration of Helsinki, and 3 ml peripheral blood was collected in EDTA vacutainers. The study was approved by the Institutional Ethics Committee of NIIH.

### Immunological Workup

Initial investigations involved a complete blood count (CBC) on a Sysmex XS-800i (Sysmex Co., Cobe, Japan) 5-part automated hematological analyzer, lymphocyte subset (LSS) analysis by flow cytometry using BD Multitest 6-color TBNK reagent followed by acquisition of cells on FACS Aria I; analysis was performed on FACS Diva and FlowJo software (BD Biosciences, San Jose, CA, USA). Serum immunoglobulin levels were estimated by Nephelometer (B N Prospec, Siemens).

Patients with normal T cells and elevated B cells were further evaluated for B cell surface markers using anti-CD24 phycoerythrin [PE], anti-CD19 allophycocyanin [APC], anti-CD38 fluorescein isothiocyanate [FITC], anti-IgD [FITC], anti-IgM peridinin–chlorophyll–protein complex: CY5.5 conjugate [PerCP-Cy5], CD10 PE Cy-7 tandem conjugate [PE-Cy7], CD5 [PerCP-Cy5], anti kappa [PE], and anti lambda [FITC] by flow cytometry.

The double negative T cells (DNT) were assessed by flow cytometric evaluation of TCR αβ^+^ PE/CD4^−^ FITC/CD8^−^ FITC, and anti-CD3 APC from BD Biosciences, San Jose, CA, USA.

### Molecular Investigations

Molecular investigations in these patients were done by Sanger sequencing. Whole exome sequencing was performed in one patient (P3) and the identified mutation was confirmed by Sanger sequencing.

#### Exome and Whole Genome Sequencing (WGS) Analysis

Genomic DNA was isolated from PBMCs of proband, parents and healthy relatives from each pedigree. Exome sequencing was generated using Sure Select Human All Exon 50Mb Kit (Agilent Technologies) coupled with Illumina HiSeq sequencing system (Illumina). WGS was generated based on Standard Coverage Human WGS from Broad Institutes. For individual samples, WES produced ~50–100× sequence coverage for targeted regions, and WGS produced 60× coverage for proband and 30× coverage for family members. DNA sequence data were aligned to the reference human genome (build 19) using Burrows-Wheeler Aligner with default parameters and variants were called using the Genome Analysis Tool Kit (3). Variants were then annotated by functional impacts on encoded proteins and prioritized based on potential disease causing genetic model. Variants with minor allele frequency <0.1% in the dbSNP (version 137), 1000 Genomes (1,094 subjects of various ethnicities; May 2011 data release), Exome Sequencing Project (4,300 European and 2,203 African-American subjects; last accessed August 2016), Ex AC databases (61,000 subjects of various ethnicities; March 2016 data release), or Yale internal database (2,500 European subjects) were filtered.

## Results

### Clinical Presentation

The median age of presentation and age at diagnosis was found to be 6 months (range: 3–36 months) and 14 months (range: 9–36 months), respectively. None of the patients belonged to consanguineous parents. All patients presented with the classical clinical findings of splenomegaly, lymphadenopathy and persistent lymphocytosis at an early age. Recurrent episodes of respiratory distress were noted in all patients except P1. *Klebsiella pneumoniae* was isolated from rectal swab of patient P2, and stool culture was positive for *Cryptosporidium* sp. Patient P3 also presented with right otitis media.

### Laboratory Investigations

**Figure 1:**
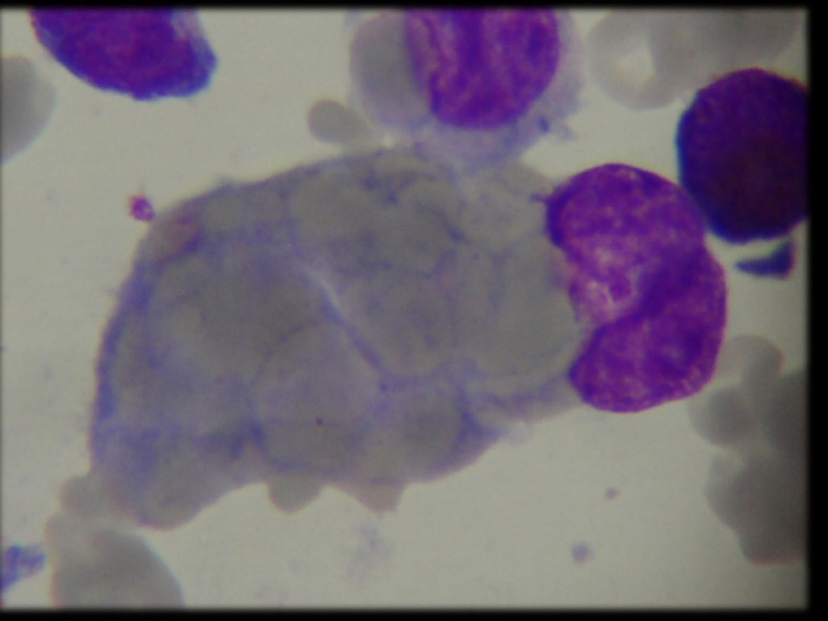
Bone marrow aspiration demonstrating hemophagocytosis in patient P4.

Complete blood count (CBC) revealed common findings of anemia, thrombocytopenia and persistent lymphocytosis in our cohort. Bone marrow aspiration and biopsy revealed trilineage hematopoiesis in all patients except P4 who presented with hemophagocytes at a later stage of illness (Figure 1). This patient also had elevated triglycerides: 311 (40–242 mg/dl), ferritin: 3,069 (7–140 ng/ml), and low level of fibrinogen: 89.00 (162–401 mg/dl) and was fitting into five out of nine diagnostic criteria for hemophagocytic lymphohistiocytosis (HLH). The infectious disease workup for all patients by serology and viral PCR including human immunodeficiency virus, hepatitis B (HbsAg), hepatitis C viruses, herpes simplex virus, cytomegalovirus, toxoplasma, rubella, and Epstein–Barr virus (EBV) was negative except in patient P1 who was positive for EBV (real-time PCR, qualitative method) at the terminal stage of illness. The autoimmune workup [antinuclear antibody (ANA), anti-double stranded DNA (ds DNA), and direct Coombs test (DCT)] revealed a positive DCT in all patients and weak positive ANA in patient P4. In view of clinical suspicion of ALPS, all patients were referred to our center for further workup. LSS revealed normal absolute T cell and NK cell counts with markedly elevated B cells. The TCR αβ^+^/CD4^−^/CD8^−^ double negative T cells (DNT) were elevated and within a range of 3.2–8.3%. B cell immunophenotyping revealed a polyclonal expansion of both immature transitional (CD10^+^CD24^hi^CD38^hi^) and mature naïve (IgD^+^) B cells. The serum immunoglobulin levels were elevated in patient P2 and within the normal range in the remaining patients. In view of hemophagocytes on BM aspirate in P4, HLH workup was performed which showed a normal perforin and granule release assay levels. The detailed immunological findings are presented in Table 1.

**Table 1:** Clinical and laboratory characteristics of B cell expansion with NF-κB and T cell anergy patients.

Patient	P1		P2		P3		P4	
Age/sex	10 months/female		1.8 months/male		9 months/female		3 years/female	
BMA/biopsy	Trilineage hematopoiesis		Trilineage hematopoiesis		Trilineage hematopoiesis		Trilineage hematopoiesis + Hemophagocytes later stage of illness	
Classical clinical manifestations: lymphadenopathy/splenomegaly/lymphocytosis	Yes		Yes		Yes		Yes	
Infections	Epstein–Barr virus (EBV) at terminal illness		Sinopulmonary		Sinopulmonary		Sinopulmonary + otitis media	
Hb (g/l)	38 (126 ± 15)		90 (125 ± 15)		78 (126 ± 15)		74 (126 ± 15)	
WBC count (×10^9^/l)	76.3 (11 ± 5)		6.8 (10 ± 5)		25.55 (11 ± 5)		28.85 (10 ± 5)	
Red Blood cell count (×10^12^/l)	1.23 (4.5 ± 0.6)		3.4 (4.6 ± 0.6)		3.35 (4.5 ± 0.6)		2.64 (4.6 ± 0.6)	
Platelet count (×10^9^/l)	86 (200–550)		3 (200–490)		165 (200–550)		300 (200–490)	
S.IgG (g/l)	6.9 (4.0–17.6)		32 (3.5–16.2)		11.8 (4.0–17.6)		12.7 (3.5–16.2)	
S.IgA (g/l)	0.06 (0.01–0.91)		3.09 (0.17–3.18)		0.07 (0.01–0.91)		0.08 (0.17–3.18)	
S.IgM (g/l)	0.43 (0.30–1.83)		3.89 (0.30–2.65)		0.43 (0.30–1.83)		1.45 (0.30–2.65)	
S.IgE (IU/ml)	312.0 (3–423)		1,190 (3–423)		35.58 (3–423)		4.45 (3–423)	
EBV Ab titers	Positive (not detected)		Negative		Negative		Negative	
Autoimmune workup	Direct Coombs test (DCT) positive ++++		DCT positive +		DCT positive +		DCT positive + antinuclear antibody positive (1:80)	

**Lymphocyte subset**	**%**	**Abs no/μl**	**%**	**Abs no/μl**	**%**	**Abs no/μl**	**%**	**Abs no/μl**

Abs lymphocytes count	94	49,000	70	17,430	87	32,294	94	3,356
CD 19^+^ B lymphocytes	79	46,060	70	12,027	81	26,150	58	1,946
CD3^+^ T lymphocytes	19	8,751	24	4,123	17	5,490	40	1,342
CD3/CD4^+^ Th lymphocytes	12	5,527	14	2,405	9	2,906	28	940
CD3/CD8^+^ Tc lymphocytes	5	2,303	8	1,374	6	1,938	8	268
CD 16^+^56^+^ NK cells	2	921	4	687	2	646	1	34
TCR αβ CD3^+^/CD4^−^/CD8^−DNT cells^	3.3	–	3.2	–	8.3	–	7.7	–
Polyclonal expansion of both immature transitional and mature naïve B cells	Yes		Yes		Yes		Yes	
*CARD11* mutation	G123S		G123D		C49Y		C49Y	

*BMA, bone marrow aspiration; Abs no, absolute number; Th, T helper cells; Tc, T cytotoxic cells; NK cells, natural killer cells; DNT, double negative T cells*.

*Age match normal range values in parentheses*.

### Genetic Diagnosis

**Figure 2:**
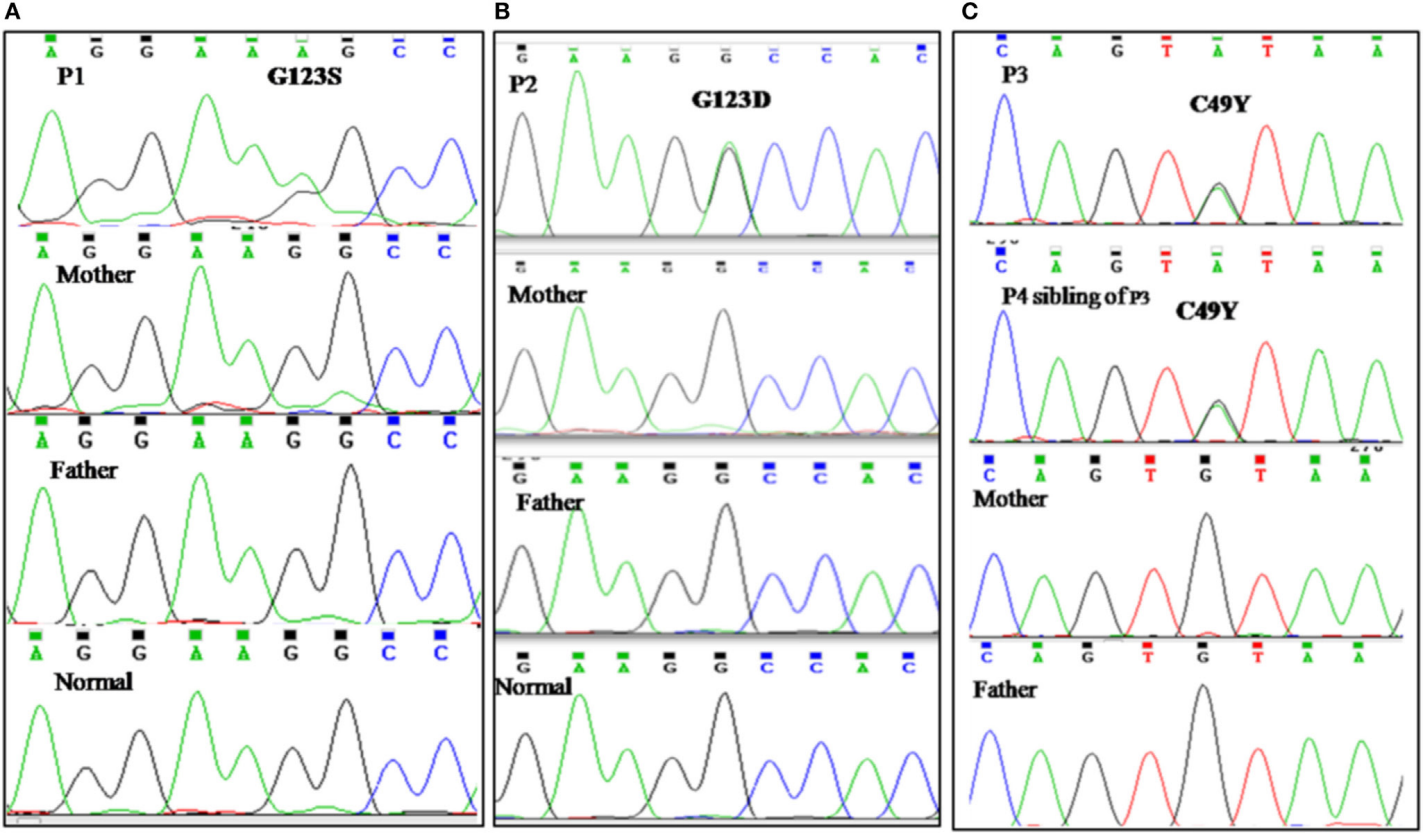
Genetic analysis. **(A)** Sanger sequencing revealed previously reported G123S mutation in *CARD 11* gene in patient P1. **(B)** Chromatogram of patient P2 showing G123D mutation in *CARD 11* gene. **(C)** P3 and P4 (sibling of P3) revealed a C49Y heterozygous, missense mutation in exon 3 of *CARD11*. Parents of all the index cases showed absence of mutation at the respective position indicating that these were *de novo* variants.

**Figure 3:**
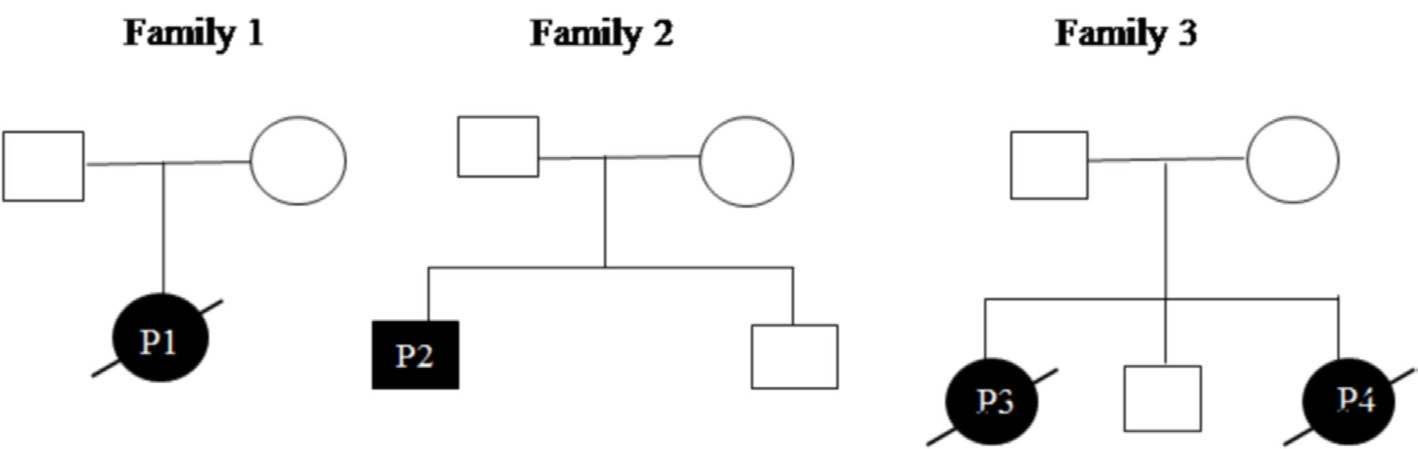
Pedigrees of the patients.

All patients were initially screened for G123S and G123D mutations of *CARD11* gene by Sanger sequencing. P1 and P2 were positive for G123S and G123D mutations, respectively. Whole exome sequencing was performed in patient P3 which revealed a heterozygous, missense mutation in exon 3 of *CARD11* (chr7: 2987283 C>T; C49Y), later confirmed by traditional Sanger sequencing. After the diagnosis of P3 by NGS, P4 (sibling of P3) was analyzed for the same mutation and was found to be positive. Parents of all the index cases showed absence of mutation at the respective position indicating that these were *de novo* variants (Figure 2). The family pedigree tree for the patients is presented in Figure 3.

### Treatment and Outcome

Patient P1 had a DCT positive anemia and was started on prednisolone 1 mg/kg for 2 weeks. However, she still required repeated hospitalizations due to severe anemia and thrombocytopenia. Later, she developed EBV infection and was restarted on steroids and other drugs including MMF, azathioprine, dapsone, and rituximab. She did not respond well and died of massive spontaneous intracerebral bleed.

Before genetic diagnosis, all our patients were strongly suspected as ALPS. Sirolimus (Rapamycin), an mTOR inhibitor is used drug for management of ALPS patients (4). In view of ALPS phenotype, patients (P2 and P3) were started on Sirolimus 1 mg/m^2^ to which both showed a good response in terms of reduction of palpable lymph nodes and splenic size. Both the patients were stable for few months. After duration of 4 months, P2 developed hypercholesterolemia. Hence, Sirolimus dose was tapered to 0.5 mg/m^2^ and then temporarily discontinued. He developed severe thrombocytopenia (3 × 10^9^/l) so he was started on Prednisolone 4 mg/kg which was gradually tapered as his platelet count normalized (300 × 10^9^/l). Presently, the child is doing well on Sirolimus 0.7 mg/m^2^. Patient P3 also responded initially to Sirolimus; however, the patient was non compliant and discontinued the treatment. She was lost to follow-up and expired due to high grade fever and respiratory distress.

In view of strong clinical suspicion of HLH in patient P4, she was started on HLH 2004 protocol consisting of dexamethasone, cyclosporine, and etoposide. However, patient did not respond and expired at the age of 3.5 years.

## Discussion

The first case of gain-of-function *CARD11* mutation was described in 2012 by Snow et al., and later in 2014 they coined the moniker BENTA disease (2, 5). Up until now, only 16 BENTA disease patients with five different germline heterozygous missense mutations (G123S, E127S, G123D, H234L, and C49Y) have been reported in the literature (6). There is a paucity of data on the clinical spectrum of BENTA disease. Here, we are reporting four cases of BENTA disease with severe clinical manifestations for the first time from India.

All patients had recurrent respiratory tract infection; however, organisms could not be isolated on repeated blood culture. One patient showed EBV positivity at the time of severe clinical deterioration. All patients were empirically treated with antibiotics to which they initially responded well. However, a severe downhill course with high grade fever was noted in all patients except P2 who required ventilator support at the initial stage of illness but, recovered later. Patient P4 showed a clinical presentation of HLH at the terminal stage of illness. All patients except P2 expired within 3 years of age confirming the severity of this disease in our series. However, the factors responsible for this clinical deterioration remain unknown. Severe autoimmune manifestations and autoantibodies have not been previously reported in BENTA patients. However, all our patients exhibited significant autoimmunity with DCT + autoimmune hemolytic anemia along with immune thrombocytopenia in two patients (P1 and P2). In addition to this, other autoimmune manifestations like “hives” was noted in P4 which is commonly observed in ALPS patients. Recently, Arjunaraja et al. (7) have shown that plasmablast/plasma cell generation and antibody production is impaired in BENTA patients. However, serum immunoglobulin levels were normal in three patients except P2 who had elevated levels.

Recently, BENTA disease has been classified under the category of a predominantly antibody deficiency by IUIS. It has been previously reported that BENTA disease is associated with T cell anergy and patients have an increased susceptibility to opportunistic viral infections like molluscum contagiosum, BK virus and Epstein–Barr virus (2, 8) as also observed in patients with combined T and B cell immunodeficiency. Also, the pattern of infection in our patient P1 and the immunoglobulin levels in all our patients do not fulfill the criteria for antibody deficiency. Hence, we feel it would be more appropriate to consider BENTA disease under the category of “Immunodeficiencies affecting cellular and humoral immunity” or “diseases of immune dysregulation.”

Genetic analysis in our patients revealed the previously reported G123S, G123D, and C49Y mutations in *CARD11* gene. The CARD11 protein is a scaffolding protein required for the transmission of signals from antigen receptors into the cell for NF-κB activation in both B and T lymphocytes. It contains an N-terminal CARD domain, a central coiled-coil (CC) domain, and a C-terminal region containing a PDZ, an SH3, and a GUK.

All the reported gain-of-function mutations are heterozygous missense mutations that mainly reside within the CC or LATCH domain of CARD11 protein and few are also noted in the CARD domain.

G123S GOF *CARD11* mutation has been described previously in two cases with classical clinical manifestations and, in one case, with mild autoimmunity. However, patient P1 with this mutation in our series had only autoimmunity without any history of infections except for EBV positivity at the terminal stages of illness. Brohl et al. have speculated that EBV could be a contributing factor to persistent lymphocytosis and high B cell numbers (5). An elevation in CD8^+^ T cells and NK cell numbers with the NKT cell numbers being under the detection limit may be associated with EBV viremia in GOF CARD11 mutation (9). We however, did not observe this in our patient as he had normal percentages and absolute numbers of CD8^+^T cells and NK cells with a detectable percentage of NKT cells.

Recently, a 20-year follow-up of patient with G123S mutation has been reported which highlighted that lymphocytosis due to a GOF *CARD11* mutation is not always detectable at early age (9). However, in contrast to this report, our patient with this mutation had lymphocytosis at early age and expired at an early age.

G123D mutation has been previously associated with profound B cell lymphocytosis (Abs count 48,262/μl) (2). Our patient with this mutation showed mild elevation of B cells (Abs count 12,067/μl) with recurrent sinopulmonary infection.

Interestingly, although the G123S and G123D mutations lie within the LATCH domain of the CARD11 protein, the single amino acid difference within the same codon was associated with a different clinical presentation in the affected patients.

C49Y mutation resides outside the LATCH-CC region of the CARD11 protein and has been previously reported in four adult patients with milder disease and mild lymphocytosis as compared with other BENTA cases. This mutation was identified in both the siblings P3 and P4 but, was absent in their parents which may arise due to germline mosaicism. Mild lymphocytosis was noted in patient P4 but P3 patient showed marked lymphocytosis. Both the patients showed elevated DNT cells (8.3 and 7.7%, respectively) compared with other BENTA patients and expired at a very early age (3 years) due to severe disease.

Both clinical and immunological overlap has been observed between ALPS and BENTA disease. Elevated DNT is observed in both the diseases and hence, cannot be considered as a hallmark of ALPS. All our patients showed elevated DNT cells (3.2–8.3% of TCR αβ T cells). However, in patients with BENTA disease, absolute T cell counts remain in normal range whereas elevated T cell counts are observed in patients with ALPS (5). Moreover, T cells in BENTA are found to be typically hypo-responsive to *in vitro* stimulation, with impaired proliferation and IL-2 secretion which is not observed in ALPS (10). B cell lymphocytosis is also common in ALPS and BENTA. However, the degree of B cell lymphocytosis is much higher in patients with BENTA disease with elevated CD10^+^CD24^hi^CD38^hi^ transitional B cells and CD19^+^CD20^+^CD5^int^ with IgD^hi^ naive mature cells population. In contrast to this, B-cells of ALPS patients show only mild elevation of IgD^hi^ naive mature and CD19^+^CD20^+^CD5^int^ population. Similar observation was noted in all our patients.

Patients with BENTA disease are generally treated with minimal intervention and by monitoring the recurrent infections the oligoclonal or monoclonal B cell expansion. It has been observed that BENTA patients have a higher chance of being predisposed to B cell malignancy, although the *CARD11* mutation alone is not responsible for B cell transformation. Newer options to specifically target the activity of CBM signaling pathway and drugs under investigation for the treatment of certain DLBCL like lenalidomide and MALT1 protease inhibitors may prove more potent than the current B-cell-depleting agents like rituximab or other general immunosuppressive drugs. Notably, in P1 lymphocytosis worsened after administration of steroids which was seen consistently on two occasions. Hence, one should suspect BENTA disease if a patient has lymphocytosis in spite of administration of immunosuppressive therapy. Patient P2 and P3 were started on Sirolimus which is a potent mTOR inhibitor. Though, P2 responded well, patient P3 who responded initially expired later due to respiratory failure. It has been previously reported that CARD11 directly activates mTOR *via* TCR/BCR (11). It will be interesting to study the clinical benefit of Sirolium in BENTA patients by determining if mTORC1 is hyperactivated in these patients causing BENTA disease.

## Conclusion

B cell expansion with NF-κB and T cell anergy patients have clinical manifestations that overlap with ALPS along with marginally elevated double negative T cells (DNT). Patients with persistent lymphocytosis and splenomegaly with or without recurrent infections should not only be suspected for ALPS but also for BENTA disease. One should suspect BENTA disease in clinical scenario of autoimmunity with or even without recurrent infections.

In our experience, extensive B cell immunophenotyping provides a clue to underlying BENTA disease which can be confirmed by genetic analysis of *CARD11* gene. Direct sequencing of reported mutations can be considered as a first line approach for rapid diagnosis. However, in unresolved cases, whole exome sequencing can be performed as a second line of approach.

## Ethics Statement

The four patients described in this report were diagnosed with BENTA disease at National Institute of Immunohaematology (NIIH). Informed consent for participating in the study was procured from the family members in accordance with the declaration of Helsinki, and 3 ml peripheral blood was collected in EDTA vacutainers. The study was approved by the Institutional Ethics Committee of NIIH.

## Author Contributions

MG and JA analyzed the data and wrote the manuscript. MG, JA, AD, SM, and MK were involved in performing laboratory investigations of the different cases. MD supervised the clinical care of the patients. ML referred two cases and provided the clinical details. PT helped in the collection of samples and the clinical details. ML and JB provided the genetic data of P3 patient. PK and MG involved in molecular analysis of patients. MM supervised the study and reviewed the manuscript.

## Conflict of Interest Statement

The authors declare that the research was conducted in the absence of any commercial or financial relationships that could be construed as a potential conflict of interest.
